# Identification of Tissue-Specific Protein-Coding and Noncoding Transcripts across 14 Human Tissues Using RNA-seq

**DOI:** 10.1038/srep28400

**Published:** 2016-06-22

**Authors:** Jinhang Zhu, Geng Chen, Sibo Zhu, Suqing Li, Zhuo Wen, Bin Li, Yuanting Zheng, Leming Shi

**Affiliations:** 1 Center for Pharmacogenomics, School of Pharmacy, and State Key Laboratory of Genetic Engineering and MOE Key Laboratory of Contemporary Anthropology, School of Life Sciences, Fudan University, Shanghai 201203, China; 2 Collaborative Innovation Center for Genetics and Development, Fudan University, Shanghai 200438, China; 3 College of Chemistry, Sichuan University, Chengdu 610064, China

## Abstract

Many diseases and adverse drug reactions exhibit tissue specificity. To better understand the tissue-specific expression characteristics of transcripts in different human tissues, we deeply sequenced RNA samples from 14 different human tissues. After filtering many lowly expressed transcripts, 24,729 protein-coding transcripts and 1,653 noncoding transcripts were identified. By analyzing highly expressed tissue-specific protein-coding transcripts (TSCTs) and noncoding transcripts (TSNTs), we found that testis expressed the highest numbers of TSCTs and TSNTs. Brain, monocytes, ovary, and heart expressed more TSCTs than the rest tissues, whereas brain, placenta, heart, and monocytes expressed more TSNTs than other tissues. Co-expression network constructed based on the TSCTs and TSNTs showed that each hub TSNT was co-expressed with several TSCTs, allowing functional annotation of TSNTs. Important biological processes and KEGG pathways highly related to the specific functions or diseases of each tissue were enriched with the corresponding TSCTs. These TSCTs and TSNTs may participate in the tissue-specific physiological or pathological processes. Our study provided a unique data set and systematic analysis of expression characteristics and functions of both TSCTs and TSNTs based on 14 distinct human tissues, and could facilitate future investigation of the mechanisms behind tissue-specific diseases and adverse drug reactions.

Many genes have numerous splice variants, promoters and protein products. Determining how the selection and diversity of isoforms is regulated requires measuring changes in the expression of individual transcripts^1^,^2^. The transcriptome is the complete set of transcripts in a cell, and their abundance under a specific development stage or physiological condition. Understanding the transcriptome is essential for interpreting the functional elements of the genome and revealing the molecular constituents of cells and tissues, and also for understanding development and disease^3^,^4^.

Different transcripts are expressed in diverse tissues or cell types, as well as in different developmental stages or diseases. With the development of next-generation sequencing, researchers can measure gene expression in various tissues at genome-wide scale^5^,^6^,^7^,^8^. Pathology caused by defects in human transcripts is usually highly tissue-specific. For example, tissue specificity of Parkinson disease, muscular dystrophy syndromes, and cardiomyopathies have been identified^9^,^10^,^11^. Such studies have found an extensive list of tissue-specific molecular pathways, both known and unexpected, that might be disrupted in disease^12^,^13^,^14^,^15^.

Noncoding RNAs (ncRNAs) are involved in many biological processes and are increasingly seen as important regulatory molecules. They regulate gene expression via sequence-specific interactions with regulatory regions at the level of transcription, RNA processing, and translation. There are different types of noncoding transcripts including long noncoding RNAs, short noncoding RNAs, pseudogenes, and so on. microRNAs, the best-studied class of short ncRNAs, mainly regulate gene expression post-transcriptionally. Long noncoding RNAs (lncRNAs) are emerging as important regulators of tissue physiology and disease process. Previous studies suggested that lncRNAs are expressed in a highly tissue-specific manner more than protein-coding transcripts are^16^,^17^,^18^,^19^. The emergence of high-throughput RNA-seq technology provides a revolutionary tool for systematically identifying diverse types of transcripts including both protein-coding and noncoding RNAs^20^.

Many published studies have investigated the nature of tissue-specific genes/transcripts on different species. For example, the Mouse ENCODE Consortium has gained great insights into both shared and species-specific transcriptional and cellular regulatory programs throughout the mouse genome in diverse cell and tissue types^21^. The SEQC consortium also described the landscape of tissue-specific gene expression based on 320 RNA-seq from 11 organs of both sexes of rats^22^. Public data sets such as the Illumina Human BodyMap 2.0 data set^18^ and more recently the Genotype-Tissue Expression Project (GTEx) data set^23^,^24^ included expression profiles of many different human tissue types, providing unique opportunities to comprehensively characterize the human transcriptomes across tissues. However, the diversity of human tissue types makes it unrealistic for a single data set to include all tissues (Supplementary Fig. S1). Although more and more studies found that noncoding RNAs play important roles in diverse physiology and disease processes, few studies comprehensively compared the expression characteristics of tissue-specific noncoding transcripts with those of tissue-specific protein-coding transcripts.

In our study, we deeply sequenced 14 human tissues including 10 types of important solid organ tissues and 4 types of important immune cells. Six of them are not included in the GTEx or Illumina Human BodyMap 2.0 data set. We found that testis expressed the highest numbers of TSCTs and TSNTs. Brain, monocytes, ovary, and heart expressed more TSCTs, whereas brain, placenta, heart, and monocytes expressed more TSNTs than the other nine tissues. A co-expression network was constructed and hub transcripts were selected based on the TSCTs and TSNTs. Each hub TSNT was co-expressed with several TSCTs. Many important tissue-specific biological processes and KEGG pathways were enriched with TSCTs. These TSCTs and the enriched biological processes closely associated with the specific functions or diseases of each tissue.

## Results

### RNA sequencing quality was high and the expression profiles of the two technical replicates were consistent

**Figure 1:**
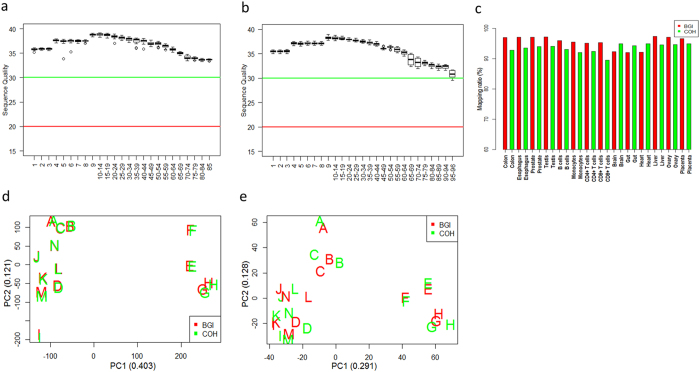
Sequencing quality check and principal component analysis. Boxplots show the sequencing quality of BGI reads (**a**) and COH reads (**b**) X axis is the base position at each read. Y axis is sequence quality score. The median of the sequencing quality score less than 20 means that sequencing quality was bad, and higher than 30 means that squencing quality was good. Mapping ratio of each sample (**c**) red and green bars represent samples sequenced at BGI and COH, respectively. Principal component analysis based on the protein-coding (**d**) and noncoding (**e**) transcripts. Principal component analysis based on the log2 (FPKM + 0.1).

In order to increase the reliability of the RNA sequencing data under the restriction of lack of biological replicates, we sequenced each tissue at two different sites, City of Hope (COH) of the United States and Beijing Genomics Institute (BGI) of China based on the Illumina platform, yielding two technical replicates for each tissue. After trimmed adapters and some low-quality reads, the overall quality of the remaining RNA-seq reads was examined using the package FastQC ( http://www.bioinformatics.babraham.ac.uk/projects/fastqc/). The medians of the sequence quality scores of all the reads across all the samples were above 30 and all samples passed quality check (Fig. 1a,b), indicating that the sequence quality was high. The trimmed high-quality reads were used for further data analyses. Mapping and quantification were conducted using the standard pipelines of TopHat and Cufflinks based on the human reference genome annotation file of Ensembl release 60 of GRCh37/hg19 that includes 52,465 genes and 157,480 transcripts^25^. On average, the mapping ratio across all the 14 tissues was high (94.6%, Fig. 1c).

After filtering lowly expressed transcripts based on the threshold of geometric mean of (FPKM + 0.1) < 1 for all 14 tissues, 26,382 transcripts were retained. Among them, 24,729 (93.7%) were protein-coding transcripts, and 1,653 (6.3%) were noncoding transcripts. Principal component analysis was performed based on log2 (FPKM + 0.1) values (Fig. 1d,e). We found that the expression profiles of the two technical replicates from the same tissue were highly consistent. Besides, Spearman’s correlation coefficients were calculated between the two technical replicates for all the 14 tissues based on protein coding (Supplementary Fig. S2) and noncoding (Supplementary Fig. S3) transcripts. The correlations ranged from 0.928 to 0.961 based on protein-coding transcripts and from 0.809 to 0.919 based on noncoding transcripts. These results further indicate high quality of experimental data.

### Eleven types of noncoding transcripts were identified

**Figure 2:**
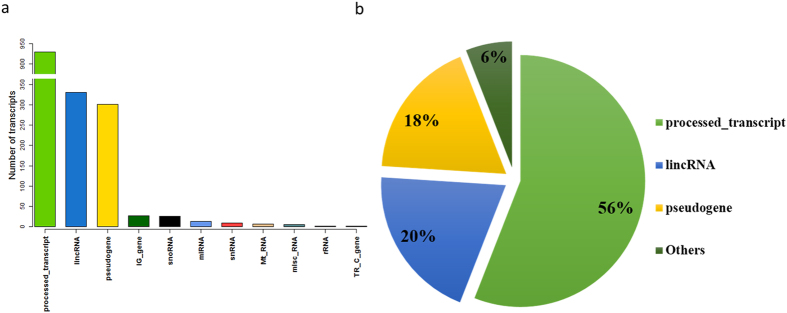
Number and percetange of transcripts in each noncoding transcript type. (**a**) Number of noncoding transcripts in each type. (**b**) Percentage of noncoding transcripts in each type. Noncoding transcript type with small relative percentages are grouped together as “Others”.

Because many noncoding transcripts including pseudogenes have polyadenylation sites, a large number of noncoding transcripts have been selected during the standard polyA selection protocol for RNA-seq used in this study^26^,^27^,^28^. In addition to protein-coding transcripts, 11 types of noncoding transcripts were annotated according to the Ensembl annotation^29^. The number and percentage of each noncoding transcript type were calculated, and most of the noncoding transcripts were processed transcripts, long intergenic noncoding RNAs (lincRNAs), and pseudogenes (Fig. 2). Processed transcripts, which do not contain an open reading frame (ORF) compared with protein-coding transcripts, showed the maximum number of 930 (56%), followed by lincRNAs (330 or 20%) and pseudogenes (301 or 18%). The number of transcripts in the other eight noncoding transcript types ranged from 2 to 27, and the total number of transcripts in these eight types was 92 (6%). All of these 1,653 annotated noncoding transcripts were used in the further analyses.

### Numbers of expressed protein-coding and noncoding transcripts vary greatly across different tissues

**Figure 3:**
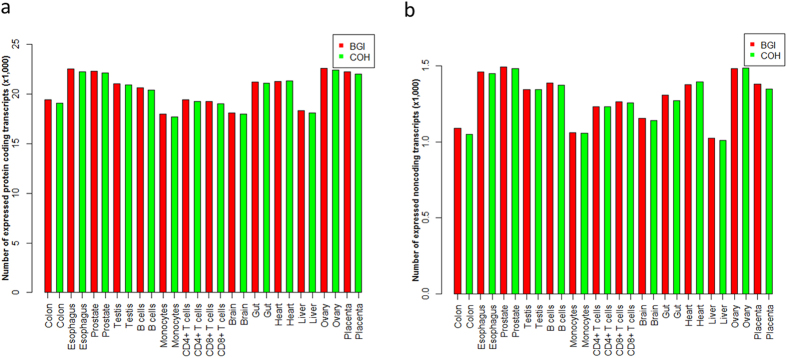
Numbers of protein-coding and noncoding transcripts expressed in the 14 human tissues. Numbers of protein-coding (**a**) and noncoding (**b**) transcripts expressed in the 14 human tissues. X axis is the samples; red and green bars represent samples sequenced at BGI and COH, respectively. Y axis is the number of expressed transcripts (×1,000). Expressed transcript detection was based on the raw FPKM values.

To detect the expressed transcripts, we defined a transcript as expressed when its original FPKM ≥ 1 in one sample. The numbers of expressed transcripts from the two technical replicates for the same tissue were consistent (Fig. 3). The numbers of expressed transcripts across all the tissues were different. Based on protein-coding transcripts, the numbers of expressed transcripts ranged from 17,696 to 22,599. Ovary expressed the largest number of transcripts. Esophagus, prostate, and placenta expressed more transcripts than the rest tissues. Monocytes expressed the least number of transcripts, and brain and liver expressed fewer transcripts than the other tissues (Fig. 3a). Based on noncoding transcripts, the numbers of expressed transcripts ranged from 1,008 to 1,494. Prostate expressed the largest number of transcripts, and ovary and esophagus expressed more transcripts than the rest tissues. Liver expressed the least number of transcripts, and monocytes and colon expressed fewer transcripts than the other tissues (Fig. 3b). On average, 20,362 protein-coding and 1,283 noncoding transcripts were expressed in each sample.

### Expression profiles of protein-coding and noncoding transcripts across 14 tissues

**Figure 4:**
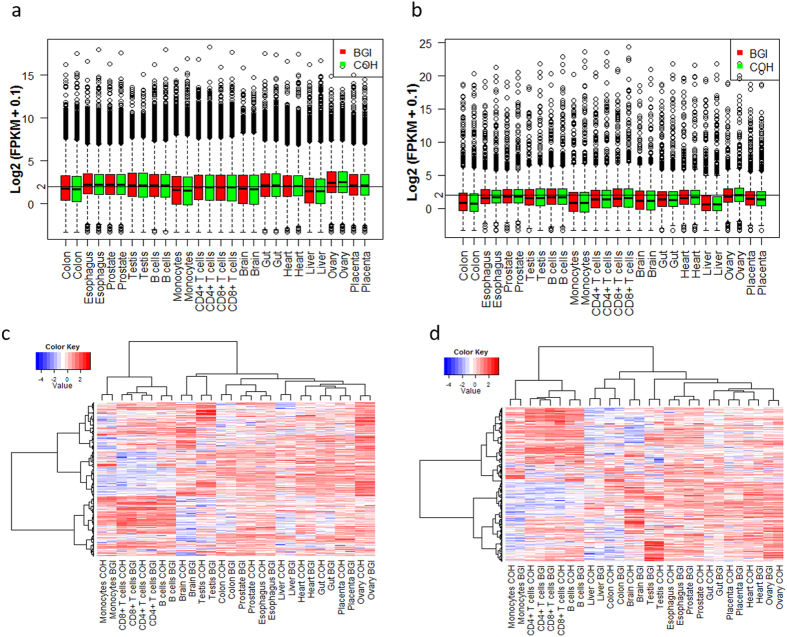
Overview of expression profiles of protein-coding and noncoding transcripts in 14 human tissues. Boxplots show the overview of expression profiles of protein-coding (**a**) and noncoding (**b**) transcripts in 14 human tissues. X axis is the samples. Red and green boxes represent samples sequenced at BGI and COH, respectively. Y axis is the log2 (FPKM + 0.1) values. Hierarchical clustering analysis based on protein-coding (**c**) and noncoding (**d**) transcripts groups tissues of similar nature together. The intensity of the color scheme is scaled to the log2 (FPKM + 0.1) expression values that are Z-score standardized per transcript in (**c**,**d**), and blue and red represent low and high expression levels, respectively.

The expression profiles of the 24,729 protein-coding and 1,653 noncoding transcripts were analyzed using boxplots and hierarchical cluster analysis (Fig. 4). We found that the overall expression levels of protein-coding transcripts were higher than those of noncoding transcripts (Fig. 4a,b). Hierarchical clustering of the tissues based on protein-coding and noncoding transcripts showed that immune cells clustered tightly as a group and tissues from solid organs clustered tightly in both protein-coding and noncoding transcripts as another group. In addition, testis and brain showed the most divergent expression profiles among all solid tissues. Monocytes showed the most divergent expression profiles among the 4 immune cells. Interestingly, prostate and esophagus clustered more tightly based on both protein-coding and noncoding transcripts, indicating that the overall expression levels of the transcripts were more similar in these two tissues (Fig. 4c,d).

### Testis expressed the largest numbers of TSCTs and TSNTs

**Figure 5:**
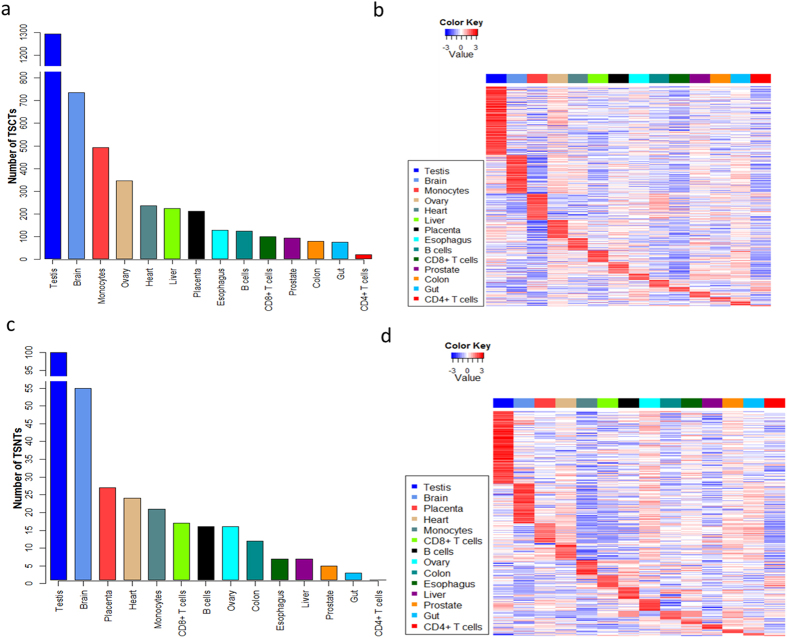
Expression profiles of TSCTs and TSNTs. (**a**) Number of TSCTs based on FC ≥ 2. X-aixs is the tissue type and Y-axis is the number of TSCTs. (**b**) Expression profiles of TSCTs. Red indicates higher expression, and blue indicates lower expression. (**c**) Number of TSNTs based on FC ≥ 2. (**d**) Expression profiles of TSNTs. Expression data are Z-score standardized per transcripts in (**b,d**). Tissue specific analysis was based on the log2 (FPKM + 0.1) values.

A threshold of FC (fold-change) ≥ 2 was used for identifying tissue-specific highly expressed transcripts. A transcript was considered as tissue-specific when its expression level in one tissue was at least two times higher than that in any other 13 tissues. TSCT analysis was performed based on the 24,729 protein-coding transcripts. We found that testis expressed the largest number of TSCTs (1,294), followed by brain (736). The numbers of TSCTs in the other 12 tissues ranged from 21 to 492 (Fig. 5a). The expression profiles of TSCTs in the 14 tissues were shown in Fig. 5b.

Tissue-specific analysis was also performed based on the 1,653 noncoding transcripts with relatively high expression values across the 14 tissues. Testis also expressed the largest number of TSNTs (100), followed by brain (55). The numbers of TSNTs in the other 12 tissues ranged from 1 to 27 (Fig. 5c). The expression levels of TSNTs in the 14 tissues were shown by the heat map (Fig. 5d).

### Biological processes and KEGG pathways significantly enriched with TSCTs reflected tissue-specific functions or involved in tissue-specific diseases of each tissue

TSCTs were involved in many important biological processes and KEGG pathways enriched by DAVID^30^. Table 1 showed some enriched biological processes and KEGG pathways for the identified TSCTs of each tissue. TSCTs of testis were significantly enriched in spermatogenesis, sexual reproduction, and some other biological processes, which were related to testis-specific functions. TSCTs of brain were significantly enriched in neuron differentiation, neuron projection development, and some other brain-specific biological processes. TSCTs of monocytes were enriched in the biological processes of immune response, inflammatory response, and so on (see Table 1 and Supplementary Table S1). Moreover, some tissue-specific pathways related to tissue-specific diseases were enriched with TSCTs. For example, Alzheimer’s disease pathway was enriched with TSCTs of brain; dilated cardiomyopathy pathway was enriched with TSCTs of heart; and autoimmune thyroid disease pathway was enriched with TSCTs of B cells (see Table 1 and Supplementary Table S2). These results suggest that TSCTs are highly related to or may exert specific functions in certain tissue-specific diseases of each tissue.

### Fourteen co-expression modules were constructed based on all the TSNTs and TSCTs

**Figure 6:**
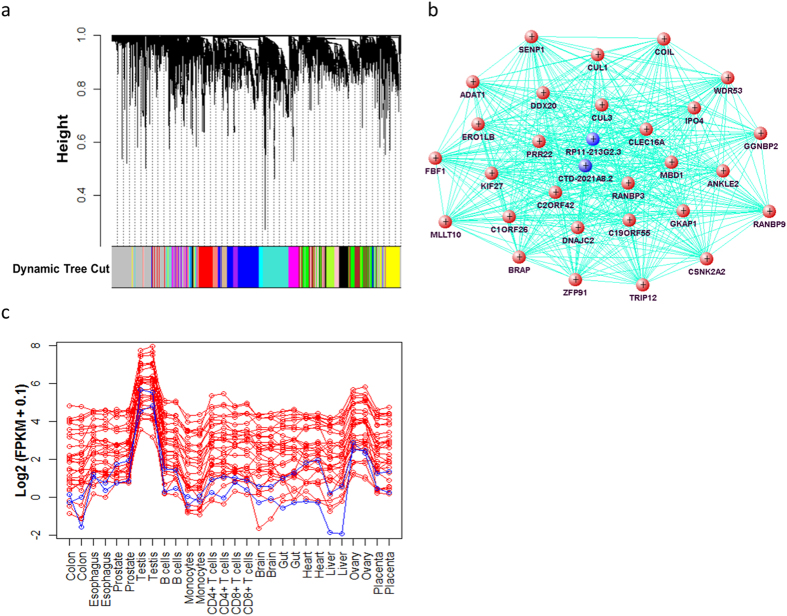
Co-expression network based on the TSNTs and TSCTs. (**a**) Clustering dendrogram based on the dissimilarity of expression profiles of all 4,471 TSNTs and TSCTs. Each colored bar (below) of the dynamic tree cut represents each module. (**b**) Network of the 30 most highly connected transcripts in the brown module. Each node is one transcript, represented by the gene name corresponding to the transcript. The 2 blue nodes correspond to 2 TSNTs, and the other 28 nodes are 28 TSCTs. Each edge represents the topological overlap or interconnectedness between two nodes. (**c**) Expression patterns of the 30 hub transcripts. Red lines are the expression levels of the 28 TSCTs and blue lines are the expression levels of the 2 TSNTs. X-axis is the sample names, and Y-axis is log2 (FPKM + 0.1) values.

To gain insight into the interaction between TSNTs and TSCTs, we constructed a co-expression network based on all of the 4,471 TSNTs and TSCTs using weighted gene co-expression network analysis (WGCNA)^31^. VisANT was used to visualize the network of hub transcripts (highly connected transcripts) of each module^32^. In addition to the gray module consisting of 956 TSNTs and TSCTs of various nature, fourteen modules of highly correlated transcripts were identified in the co-expression network (Fig. 6a). Each module represented a group of transcripts with similar expression profiles across all the samples. The biggest module consisted of 549 TSNTs and TSCTs. The number of transcripts in the second biggest module was 501. The transcript numbers of the remaining 12 modules ranged from 87 to 297. The regulatory functions of TSNTs with TSCTs could be accomplished by constructing a co-expression network.

Through further analysis of co-expression network between TSNTs and TSCTs of each module, many hub TSNTs were found highly co-expressed with several TSCTs. Take the brown module as an example. Thirty (30) hub transcripts (2 TSNTs and 28 TSCTs) of this module for testis were selected (Fig. 6b). The pairwise Pearson correlation coefficient between the expression profiles of these 30 hub transcripts ranged from 0.72 to 1. Grouping of highly correlated transcripts could be a result of transcriptional co-activation or the co-regulation of mRNA stability. Hence, these 2 TSNTs may regulate the expression level of the 28 TSCTs. Figure 6c was the co-expression pattern of these 30 hub transcripts. We found that these 30 transcripts were highly correlated and highly expressed in testis. Intriguingly, these 30 testis-specific highly expressed transcripts were also expressed a little bit higher in ovary than in the other 12 tissues (Fig. 6c). Therefore, this finding further demonstrated that these 30 testis-specific highly expressed transcripts may indeed have sex-specific functions. Furthermore, some of these 30 testis-specific transcripts/genes have been reported to be related with some testis-specific functions or diseases. For example, CSNK2A2 was found preferentially expressed in late spermatogenesis, and male mice with disrupted CSNK2A2 were infertile with oligospermia and globozoospermia^33^. GKAP1 gene encodes a protein that is highly similar to the mouse cGMP-dependent protein kinase anchoring protein 42 kDa. The mouse protein has been found to localize with the Golgi and recruit cGMP-dependent protein kinase I alpha to the Golgi in mouse testis. It is thought to play a role in germ cell development^34^. These molecular biology results validated parts of our findings.

### Validation of results using the GTEx dataset

The GTEx project has sequenced the largest number of normal human tissues including 43 body sites of 27 tissues. This project aims to describe the relationship among genetic variation, gene expression, and other molecular phenotypes^23^,^24^. In total, 8 tissues were in common between GTEx and our dataset (Supplementary Fig. S1). Therefore, we could validate our results using the transcriptomic dataset of the 8 overlapping tissues of GTEx.

The same pipeline for our dataset analysis was used for analyzing the RNA-seq data of the 8 overlapping tissues of GTEx project, and some of our results were further validated. First, prostate and esophagus also expressed more protein-coding and noncoding transcripts, and liver expressed fewer protein-coding and noncoding transcripts (Supplementary Fig. S4). Secondly, the overall expression levels of protein-coding transcripts were higher than those of noncoding transcripts (Supplementary Fig. S5a,b). Thirdly, testis exhibited the most different expression profiles among the 8 tissues based on both protein-coding and noncoding transcripts (Supplementary Fig. S5c,d). Fourthly, testis expressed the most TSCTs and TSNTs (Supplementary Fig. S6). These results were largely consistent with our results, further indicating the reliability of our results.

## Discussion

We created and explored a unique data set of the transcriptomic profiles of 14 human tissues including 10 important solid organ tissues and 4 immune cell types. Because of the difficulty of acquiring human normal tissues, we were unable to obtain biological replicates for our tissues, preventing rigorous statistical analysis in our study. Therefore, only fold change was used to identify tissue-specific transcripts. We took several steps to ensure the reliability of our results. First, we deeply sequenced each of the 14 tissues at two different sites and demonstrated high level of concordance between sites. Secondly, to minimize the sensitivity of fold change calculations to transcripts with low expression^25^, we filtered low expressors with geometric mean < 1. Thirdly, our results are largely in agreement with those from our reanalysis of the GTEx dataset. In addition, our findings, e.g. testis-specific expression of transcripts/genes, have been previously reported in the literature, corroborating well with our results.

Through the tissue specific transcripts analysis, we found that testis expressed the largest number of TSCTs, followed by brain and monocytes. Similar results were reported previously based on tissues of human and other species^18^,^22^,^35^. Enrichment analysis showed that the TSCTs were involved in important tissue-specific biological processes that were highly correlated with the corresponding tissue-specific functions. For example, TSCTs of testis were most significantly involved in the sexual reproduction biological process, whereas TSCTs of brain were most significantly involved in the synaptic transmission biological process. Furthermore, some KEGG pathways related to tissue-specific diseases were enriched with TSCTs. For example, the Alzheimer’s disease pathway was enriched with TSCTs of brain, and the dilated cardiomyopathy pathway was enriched with TSCTs of heart, and so on. Therefore, these TSCTs and the enriched tissue-specific biological processes may determine the specific functions or involved in some tissue-specific diseases of each tissue. Consequently, they may be related to the tissue-specific diseases and adverse drug reactions. These results also indicated that our methods for searching TSCTs and TSNTs are reliable.

Expression of noncoding transcripts demonstrates tissue specificity and is involved in various kinds of diseases^36^,^37^,^38^,^39^. In our study, we first identified 11 types of noncoding transcripts based on the Ensembl annotation system. We also investigated the nature of the TSNTs based on these 14 human tissues. We found that testis expressed the largest number of TSNTs just as it did in protein-coding transcripts, followed by brain, placenta, heart, and monocytes. Functional annotation of noncoding transcripts has been hampered by the lack of comprehensive noncoding transcript annotation resources^19^,^40^, and we tried to annotate the functions of these TSNTs using co-expression network analysis.

Modules of transcripts with expression profiles highly correlated could result from transcriptional co-activation, the co-regulation of mRNA stability, or a combination of both, thus accomplishing a group of related functions^41^,^42^. In our study, we investigated the co-expression network between TSNTs and TSCTs. Fourteen modules were formed and each module corresponded to one tissue type. Hub transcripts were of special interest because they were the backbones of the scale-free network architecture. For example, the testis module consisted of 30 hub transcripts including 2 TSNTs and 28 TSCTs. The high correlation of expression profiles between these 30 hub transcripts indicated that these 2 TSNTs may co-activate or co-regulate the expression level of the 28 TSCTs. Therefore, we could infer that these TSNTs may play biological functions of tissue specificity through regulating the expression level of TSCTs of each tissue type.

The immune cell type specificity of expression profiles of transcripts is related to autoimmune diseases^43^,^44^,^45^. The TSCTs and TSNTs of the 4 types of immune cells were analyzed in junction with the 10 solid organ tissues in our study. We found that monocytes showed the largest numbers of TSCTs and TSNTs in these 4 types of immune cells. Many immune cell-specific biological processes and KEGG pathways related to immunological reactions were identified. For example, pathogenic *Escherichia coli* infection pathways were enriched with TSCTs of monocytes, and graft-versus-host disease and autoimmune thyroid disease pathways were enriched with TSCTs of B cells. These results could facilitate further understanding of immunological reactions and some immunological diseases.

We found that testis exhibited the most significant tissue-specific characteristics in two aspects. First, testis exhibited the most diverse expression profiles both in protein-coding transcripts and in noncoding transcripts seen through the hierarchical clustering analysis based on the 10 solid organ tissues. Secondly, we found that testis expressed the largest numbers of TSCTs and TSNTs. In our study, the testis tissue came from a male adult donor who was sexually mature. Testicular cells are different from other somatic cells and play important roles in spermatogenesis, steroidogenesis and development of male sex characteristics^46^,^47^,^48^. These results suggested that testis may need the expression of more specific transcripts for maintaining its complex functions.

In summary, we generated high quality RNA-seq data from a set of 14 diverse human tissues. We found that testis expressed both the largest numbers of TSCTs and TSNTs. Some hub TSNTs were highly co-expressed with the hub TSCTs. Important tissue-specific biological processes and KEGG pathways were enriched with TSCTs, indicating that they participated in the specific biological functions or specific diseases of each tissue. Our findings could help researchers to further investigate the mechanisms of tissue-specific diseases and adverse drug reactions in the future.

## Methods

### Cell and solid organ tissue samples

The 10 human solid tissues were collected post mortem as part of a rapid autopsy program from 2 individuals, one of them was a fetus. The 4 haematopoietic cells were isolated from leukopaks obtained from a healthy volunteer participating in routine platelet pheresis. Therefore, the 14 human tissues were from 3 individuals, denoted as A, B, and E. Samples from colon, esophagus, prostate, and testis were collected from individual A. Samples from B cells, monocytes, CD4 + T cells, and CD8 + T cells were collected from individual B. Samples from brain, gut, heart, liver, ovary, and placenta were collected from a fetus E. All of the 14 tissues were a subset of the 30 tissues studied using high-resolution Fourier–transform mass spectrometry, and the corresponding proteomic results were published in 2014 in *Nature*^49^. That study was approved by the Johns Hopkins University’s Institutional Review Board for use of human tissues and informed consent was obtained from all subjects from whom blood samples were obtained for isolation of haematopoietic cells. The methods were carried out in accordance with the approved guidelines. All the samples were histologically confirmed to be normal, and stored at −80 °C all the time. Total RNA was extracted from tissue by using the miRNeasy Mini Kit (Qiagen, Valencia, CA, USA) according to the manufacturer’s protocol. RNAs longer than 18 nucleotides were recovered with this method. RNA sample information along with RNA integrity numbers (RINs) was shown in Supplementary Table S3. In addition, the Bioanalyzer profiles of all 14 samples were provided in Supplementary Fig. S7. As can be seen, most samples are of good quality with the lowest RIN of 6.2 and the mean RIN of 8.4. We thank Prof. Akhilesh Pandey for providing us the 14 RNA tissues sequenced in our study.

### Library preparation and deep RNA sequencing

We used a polyA selection protocol coupled with the Illumina TruSeq RNA-seq library protocol to construct the human body map RNA-seq libraries. RNA-seq libraries were sequenced using Illumina’s TruSeq Cluster V3 flow cells and TruSeq SBS Kit (Illumina). The 14 human tissues were sequenced completely independently at two sites (BGI with HiSeq 2000 and COH with HiScanSQ). About 127 million pair-end reads of 90 bp were generated for each sample at BGI, and about 57 million pair-end reads of 101 bp were generated for each sample at COH.

### Mapping, quantification and primary analysis of the RNA-seq data

RNA-seq fastq raw data were trimmed to remove adapters and low quality reads using Trimmomatic v0.30^50^. The trimmed reads were mapped to the Ensembl hg19 reference genome using TopHat v2.04^51^ , allowing a maximum of 2 mismatches, and default values for all the other parameters were used. Transcripts assembly and quantification were done using Cufflinks v2.02^52^, with all default parameter settings based on the human annotation file of Ensembl GRCh37. After quantification using Cufflinks, we obtained a matrix of expression values in FPKM with 156,600 transcripts and 14 samples. Except for the detection the number of expressed transcripts where raw FPKM values were used, a value of 0.1 was added to each raw RPKM value of the expression data matrix before being transformed to log2 scale and downstream data analysis. It should be noted that the choice of a pseudo value of 0.1 added to RPKM in some of the downstream analysis is arbitrary, but consistent with previous publications favoring a relatively large FPKM pseudo value such as 1.0^22^,^35^. Among the 156,600 transcripts, 25,273 transcripts were not expressed at all in any of the 14 tissues. In addition, the average expression value (FPKM) of 67,661 transcripts (43.2%) was below 0.1. We analyzed our data sets with different pseudo values (1, 0.1, and 0.01). While large pseudo values lead to fold-change compression for low expressors as expected, using small pseudo value (e.g. 0.01 or lower) made the fold changes and the resulting lists of tissue-specific genes unstable, in line with observations in a recent reference benchmark study of RNA-Seq^53^. In that study, the issue was resolved by an additional threshold for expression strength. We here chose to employ a relatively large pseudo-count which, by attenuating fold-change values for low expressors, effectively introduces a soft thresholding for weak expressors. As a consequence, our survey has a focus clearly expressed genes. After filtering transcripts with low expression values using the geometric mean of (FPKM + 0.1) < 1 for the 14 tissues as the threshold, 26,382 transcripts remained, of which 24,729 were protein-coding and 1,653 were noncoding transcripts. The resulting data matrix of 26,382 rows by 14 columns was used to identify TSCTs and TSNTs. All the statistical analysis was conducted using R statistical programming language ( http://www.r-project.org/).

### Identification of noncoding transcripts

In the human transcriptome annotation file of Ensembl GRCh37, each transcript was classified as one of many biotypes, including protein-coding, processed, pseudogene, and so on. In our study, all the transcripts rather than protein-coding were termed as noncoding transcripts. We used in-house R scripts to classify the 1,653 noncoding transcripts according to the human annotation file. In addition, all kinds of pseudogenes (such as miRNA_pseudogene, snRNA_pseudogene, IG_pseudogene, and so on) were merged as pseudogene; all kinds of TR_gene were merged as TR_gene; all kinds of IG_gene were merged as TG_gene, and so on. At last, 11 types of noncoding transcripts were identified.

### Selection of TSCTs and TSNTs

TSCTs and TSNTs were selected using threshold of FC ≥ 2 to select the TSCTs and TSNTs. A transcript was considered as tissue-specific when its expression level in one tissue was at least two times higher than that in any other 13 tissues. After selection of TSCTs and TSNTs, 2 smaller matrices were constructed corresponding to the expression values of all the TSCTs and TSNTs. Each column represents a sample and each row represents a transcript in these 2 matrices. The transcripts were sorted according to the numbers of TSCTs and TSNTs from maximum to minimum. These 2 matrices were Z-score standardized (mean of zero and s.d. of one) per transcript. Function heatmap.2 of gplots R package was used for the heatmap drawing based on these 2 standardized matrices with the parameters of Rowv = F, Colv = F.

### Weighted transcripts co-expression network construction and module detection

The WGCNA package in R was used for step-by-step network construction and module detection. Weighted network uses soft threshold of the Pearson correlation between the expression profiles for determining the connection strengths between two transcripts. The connection strength between the expression profiles of transcripts x_i_ and x_j_ across all samples is defined as α_ij_ = |cor(x_i,_ x_j_)|^β^. We chose the soft thresholding power β = 20 based on the criterion of scale-free topology. Average linkage hierarchical clustering was performed to group transcripts based on topological overlap dissimilarity measurement of their network connection strengths. Modules were identified with a dynamic tree-cutting algorithm with a minimum module size of 60 transcripts. In our study, all of the TSNTs and TSCTs were used for constructing the co-expression network. Most of the TSNTs and TSCTs of the same tissue were found in the same module, allowing us to investigate the relationship of the TSNTs and TSCTs of each tissue. Hub transcripts were extracted using the function exportNetworkToVisANT of the WGCNA package, and their interactive network was visualized using VisANT.

### Accession codes

The RNA-seq data set of 14 human tissues has been deposited in NCBI Gene Expression Omnibus (GEO) under accession code GSE83115.

## Additional Information

**How to cite this article**: Zhu, J. *et al*. Identification of Tissue-Specific Protein-Coding and Noncoding Transcripts across 14 Human Tissues Using RNA-seq. *Sci. Rep.* **6**, 28400; doi: 10.1038/srep28400 (2016).

## Supplementary Material

Supplementary Information

## Figures and Tables

**Table 1:** Tissue-specific Gene Ontology biological processes and KEGG pathways significantly enriched with TSCTs in each tissue.

Tissue type	Biological process/KEGG pathway	P-value	Count
Testis	Spermatogenesis	1.96E-06	36
	Male gamete generation	1.96E-06	36
	Sexual reproduction	4.37E-05	43
Brain	Neuron differentiation	6.50E-10	42
	Neuron projection development	5.32E-08	28
	Alzheimer’s disease	6.82E-02	10
Monocytes	Immune response	1.02E-17	57
	Defense response	1.48E-16	52
	Inflammatory response	6.96E-12	32
Ovary	Progesterone-mediated oocyte maturation	2.78E-03	7
Heart	Heart contraction	1.54E-09	8
	Dilated cardiomyopathy	1.33E-07	11
	Hypertrophic cardiomyopathy (HCM)	7.53E-07	10
Liver	Oxidation reduction	2.35E-13	33
	Steroid metabolic process	6.01E-13	20
	Response to nutrient	1.35E-02	6
Placenta	Positive regulation of developmental process	7.04E-03	9
	Response to nutrient	1.35E-02	6
	Female pregnancy	2.49E-02	5
Esophagus	Oxidation reduction	9.42E-04	13
	Response to protein stimulus	5.40E-03	5
	Glycerolipid metabolism	5.84E-03	4
B cells	Immune response	6.50E-06	16
	Graft-versus-host disease	2.61E-02	3
	Autoimmune thyroid disease	4.27E-02	3
CD8 + T cell	Immune response	3.49E-02	8
	Positive regulation of defense response	4.28E-02	3
	Positive regulation of natural killer cell mediated immunity	6.98E-02	2
Prostate	Muscle contraction	5.31E-04	6
	Gland development	2.10E-02	4
Colon	Carbohydrate biosynthetic process	5.36E-03	4
	Response to nutrient levels	2.76E-02	4
	Response to drug	3.49E-02	4
Gut	Protein digestion	1.10E-02	2
	Multicellular organismal protein catabolic process	1.10E-02	2
	Secretion	2.46E-02	5
CD4 + T cells	Regulation of Ras protein signal transduction	3.12E-02	3
	Regulation of small GTPase mediated signal transduction	4.35E-02	3

In this table, columns 1, 2, 3, 4 represent the tissue types, the significantly enriched tissue-specific biological processes or KEGG pathways, the enriched p-values, and the numbers of TSCTs involved in the biological processes or KEGG pathways, respectively.
