# Supplementary material for: Identification of Tissue-Specific Protein-Coding and Noncoding Transcripts across 14 Human Tissues Using RNA-seq
Source: Sci Rep. 2016 Jun 22;6:28400. doi: 10.1038/srep28400 (PMC4916594; doi:10.1038/srep28400)
Supplement: Supplementary Information [file srep28400-s1.pdf]

Identification of Tissue-Specific Protein-Coding and Noncoding Transcripts across 14 Human Tissues Using RNA-seq

Jinhang Zhu[1], Geng Chen[1], Sibo Zhu[1,2], Suqing Li[3], Zhuo Wen[3], Bin Li[1], Yuanting Zheng[1,2*] and Leming Shi[1,2*]

[1]Center for Pharmacogenomics, School of Pharmacy, and State Key Laboratory of Genetic Engineering and MOE Key Laboratory of Contemporary Anthropology, School of Life Sciences, Fudan University, Shanghai 201203, China.

[2]Collaborative Innovation Center for Genetics and Development, Fudan University, Shanghai 200438, China.

[3]College of Chemistry, Sichuan University, Chengdu 610064, China.

*To whom correspondence should be addressed (yuantingzheng@fudan.edu.cn or lemingshi@fudan.edu.cn).

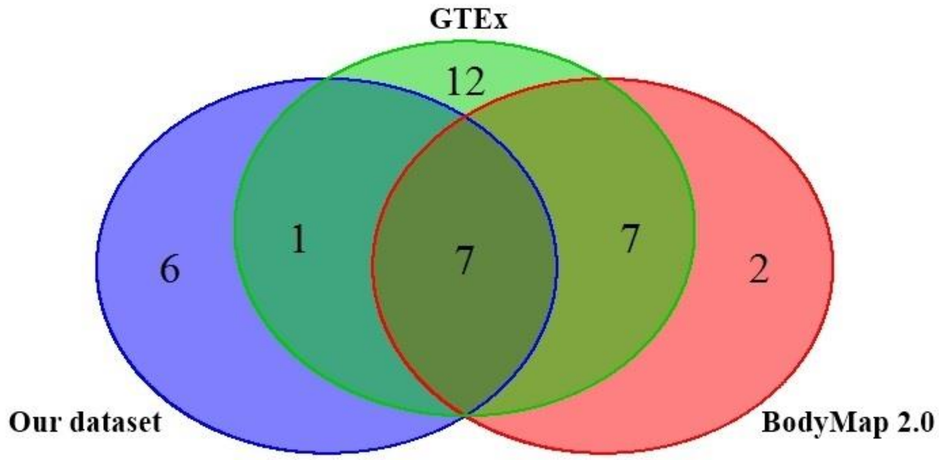

Supplementary Figure S1. Overlapping tissues among our data set, Illumina Human BodyMap 2.0 data set, and the GTEx data set. The three data sets shared seven common tissues (brain, colon, heart, liver, ovary, prostate, and testis). Eight (8) tissues (brain, colon, esophagus, heart, liver, ovary, prostate, and testis) are in common between our data set and the GTEx data set. Fourteen (14) tissues (adrenal, adipose, brain, breast, colon, heart, kidney, liver, lung, ovary, prostate, muscle, testis, and thyroid) are in common between Illumina Human BodyMap 2.0 data set and the GTEx data set.

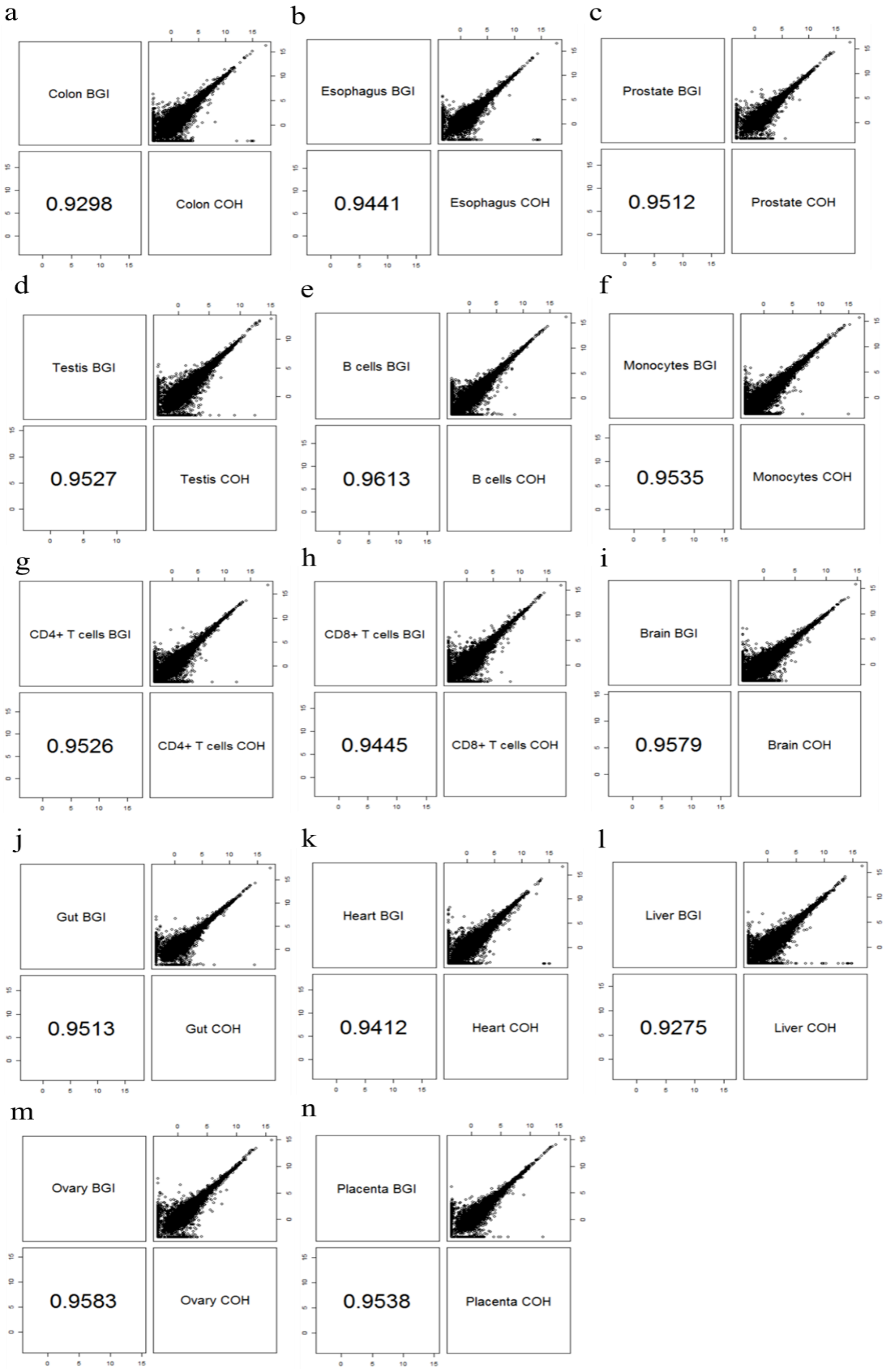

Supplementary Figure S2. Spearman's correlations and scatterplots of two technical replicates of all the 14 tissues based on protein coding transcripts.

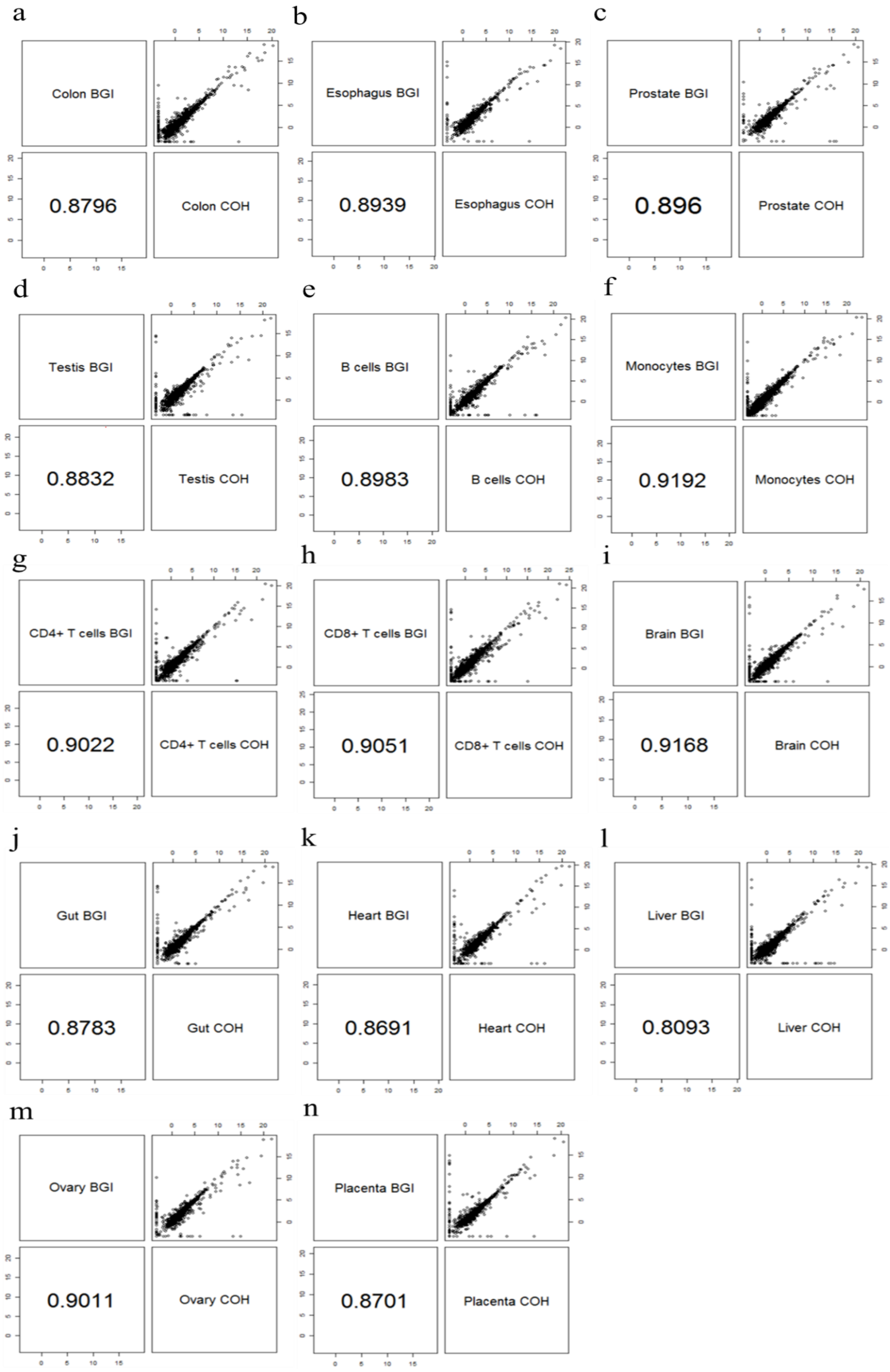

Supplementary Figure S3. Spearman's correlations and scatterplots of two technical replicates of all the 14 tissues based on noncoding transcripts.

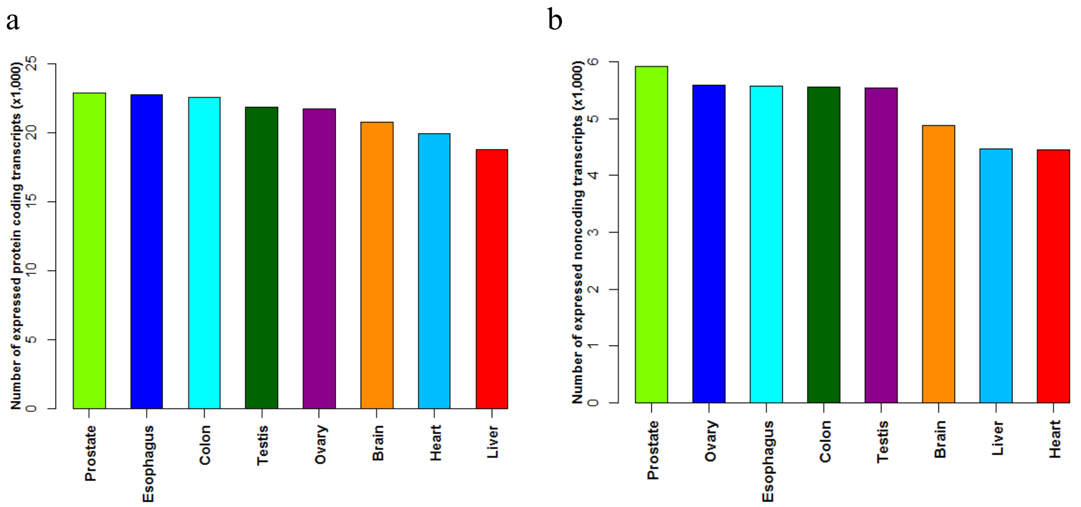

Supplementary Figure S4. Numbers of protein-coding (a) **and noncoding** (b) **transcripts expressed in the 8 overlapping tissues of the GTEx data set**. X axis is the tissues. Y axis is the number of expressed transcripts (x 1,000).

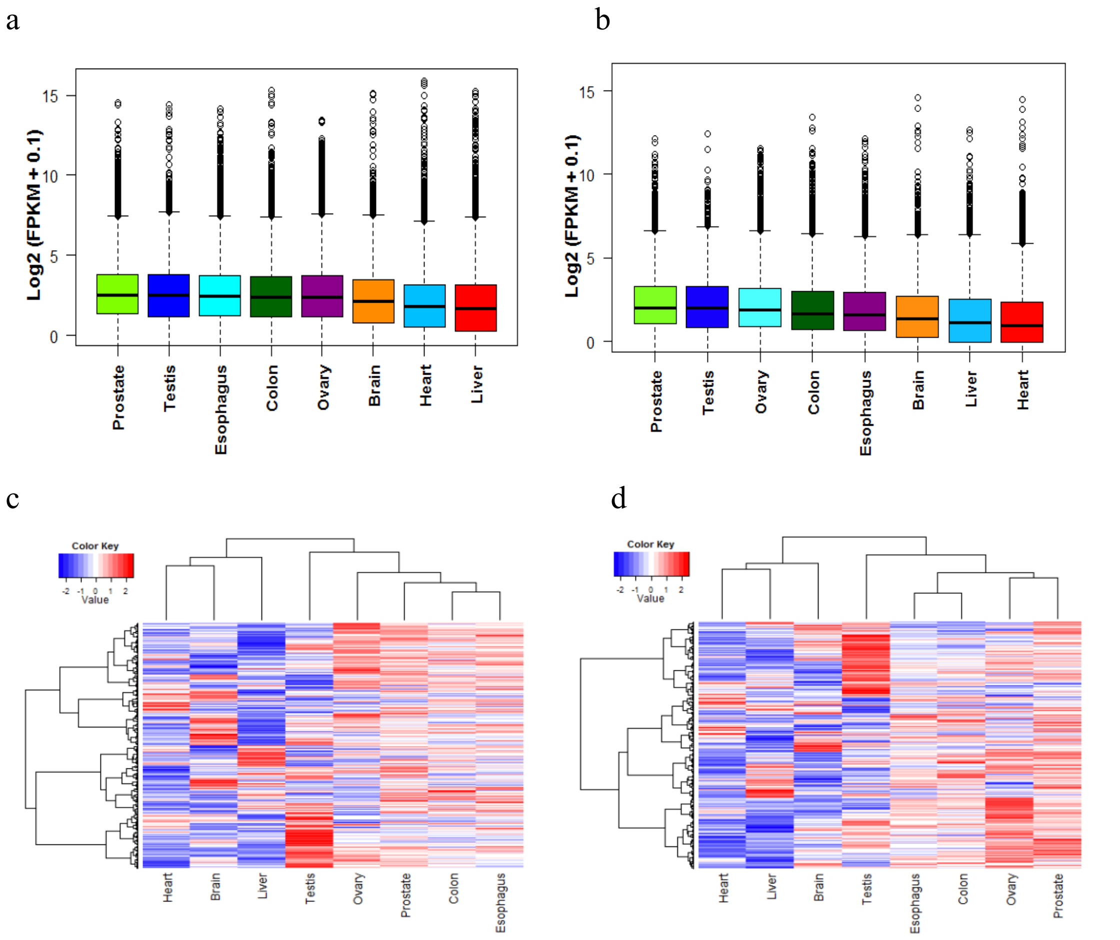

Supplementary Figure S5. Overview of expression profiles of protein-coding and noncoding transcripts in the 8 overlapping tissues of the GTEx data set. Boxplots show the overview of expression profiles of protein-coding (a) and noncoding (b) transcripts in 8 human tissues. X axis is the tissue types. Y axis is the log2 (FPKM+0.1) values. Hierarchical clustering analysis based on protein-coding (c) and noncoding (d) transcripts groups tissues of similar nature together. The intensity of the color scheme is scaled to the log2 (FPKM+0.1) expression values that are Z-score standardized per transcript in (c) and (d), and blue and red represent low and high expression levels, respectively.

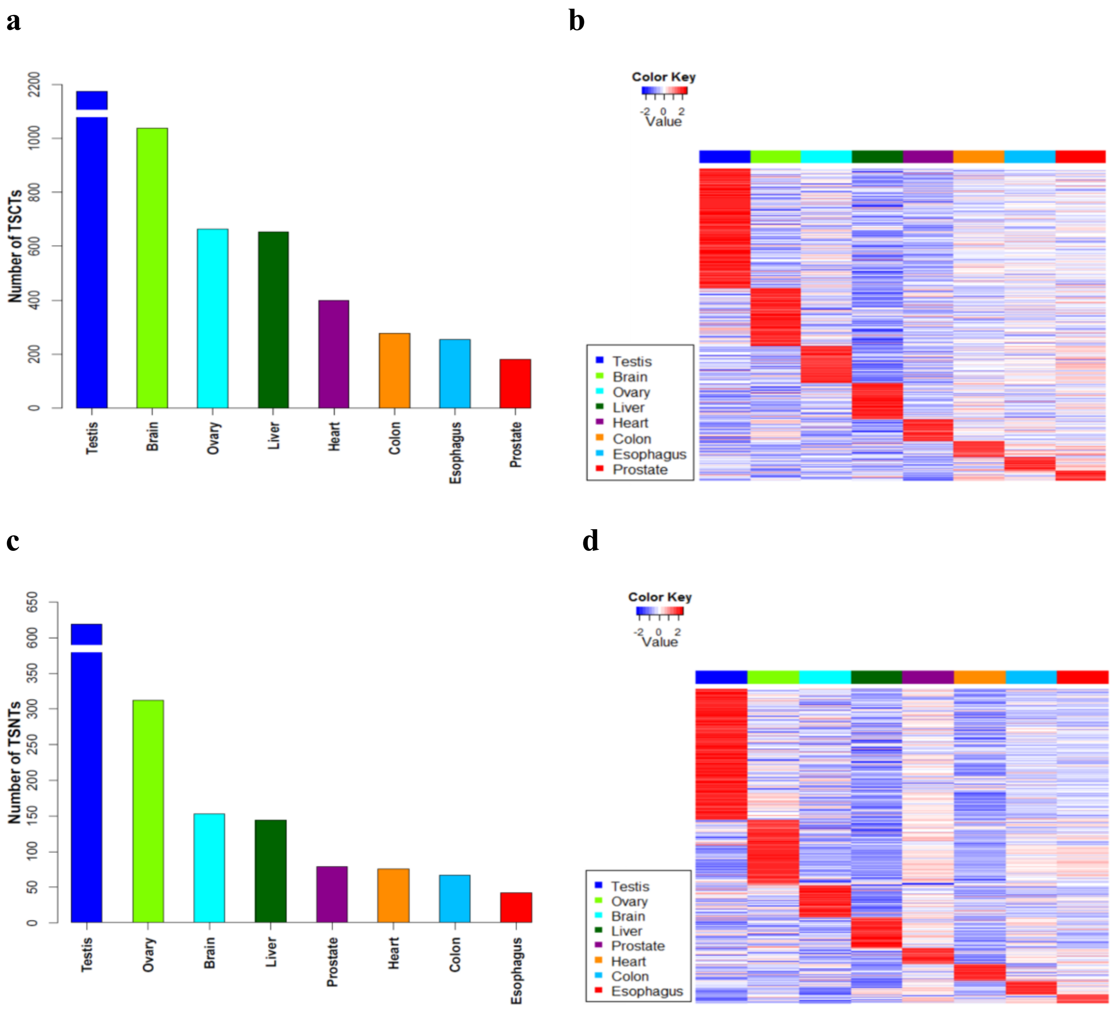

Supplementary Figure S6. Expression profiles of TSCTs and TSNTs in the 8 overlapping tissues of the GTEx data set. (a) Number of TSCTs based on FC ≥ 2. X-aixs is the tissue type and Y-axis is the number of TSCTs. (b) Expression profiles of TSCTs. Red indicates higher expression, and blue indicates lower expression. (c) Number of TSNTs based on FC ≥ 2. (d) Expression profiles of TSNTs. Expression data are Z-score standardized per transcripts in (b) and (d). Tissue-specific analysis was based on the log2 (FPKM+0.1) values.

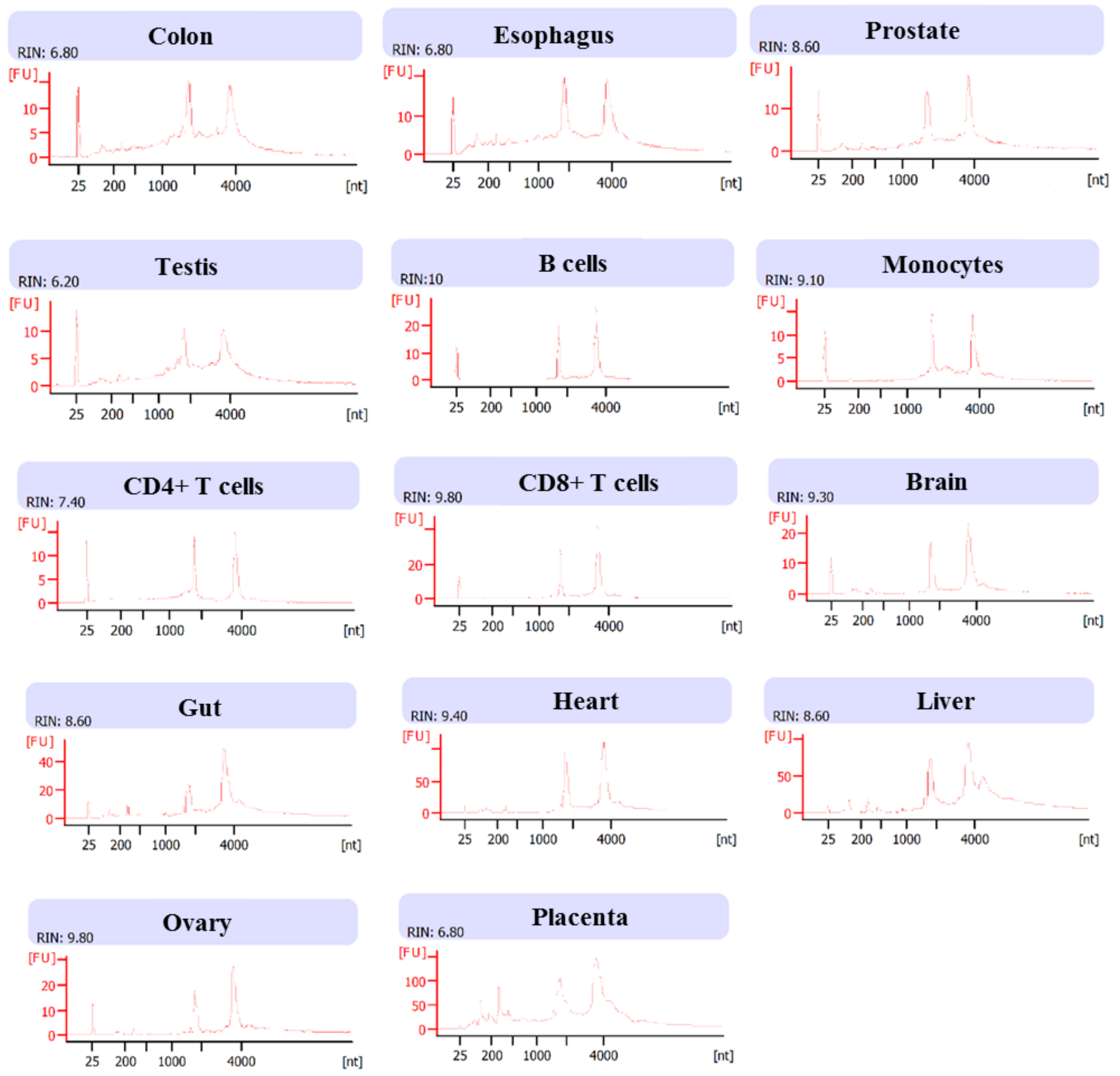

Supplementary Figure S7. Bioanalyzer profiles of the 14 human RNA samples sequenced in the study.

Supplementary Table S1. Top 10 biological processes significantly enriched with TSCTs for each of the 14 tissues. In this table, columns 1, 2, 3, 4 represent the tissue types, the top 10 most significantly enriched biological processes, the enriched p-values, and the numbers of TSCTs involved in the biological processes, respectively. All the biological processes were listed if the number of enriched biological processes under 10.

Tissue type	Biological process	P-value	Count
Testis	Spermatogenesis	1.96E-06	36
	Male gamete generation	1.96E-06	36
	Cell cycle process	2.19E-06	54
	Protein modification by small protein conjugation or removal	2.67E-05	22
	Gamete generation	3.56E-05	39
	DNA repair	4.32E-05	31
	Sexual reproduction	4.37E-05	43
	Mitotic cell cycle	4.52E-05	37
	DNA metabolic process	9.99E-05	45
	Protein targeting	1.05E-04	25
Brain	Neuron differentiation	6.50E-10	42
	Microtubule-based process	4.16E-08	28
	Neuron projection development	5.32E-08	28
	Microtubule-based movement	1.07E-07	18
	Neuron development	4.77E-07	31
	Negative regulation of cellular component organization	6.34E-07	19
	Axon cargo transport	7.69E-07	8
	Cell projection organization	2.60E-06	31
	Intracellular signaling cascade	2.71E-06	71
	Neuron projection morphogenesis	4.84E-06	22
Monocytes	Immune response	1.02E-17	57
	Defense response	1.48E-16	52
	Response to wounding	2.28E-12	42
	Inflammatory response	6.96E-12	32
	Response to bacterium	1.06E-08	21
	Taxis	2.71E-06	16
	Chemotaxis	2.71E-06	16
	Response to molecule of bacterial origin	2.96E-06	12
	Regulation of tumor necrosis factor production	4.09E-06	8
	Response to lipopolysaccharide	7.31E-06	11
Ovary	DNA metabolic process	6.65E-14	39

	Chromosome organization	9.50E-10	32
	Cellular macromolecular complex subunit organization	1.58E-09	27
	Cell cycle	5.80E-08	38
	DNA replication	6.32E-08	18
	Response to DNA damage stimulus	7.45E-08	25
	Cellular macromolecular complex assembly	7.63E-08	23
	Macromolecular complex subunit organization	1.93E-07	35
	DNA repair	2.31E-07	21
	Chromatin organization	3.87E-07	24
Heart	Heart process	1.54E-09	8
	Heart contraction	1.54E-09	8
	Generation of precursor metabolites and energy	9.83E-09	18
	Cardiac muscle contraction	1.88E-08	7
	Heart development	1.75E-07	14
	Muscle organ development	1.03E-06	13
	Cardiac muscle tissue development	1.26E-06	8
	Circulatory system process	2.02E-06	12
	Blood circulation	2.02E-06	12
	Striated muscle contraction	5.24E-06	7
Liver	Sterol metabolic process	5.61E-16	18
	Cholesterol metabolic process	2.56E-15	17
	Sterol biosynthetic process	5.70E-14	12
	Cholesterol biosynthetic process	7.71E-14	11
	Oxidation reduction	2.35E-13	33
	Steroid metabolic process	6.01E-13	20
	Lipid biosynthetic process	6.90E-13	24
	Steroid biosynthetic process	1.15E-10	13
	Neutral lipid metabolic process	2.83E-09	10
	Acylglycerol metabolic process	4.97E-08	9
Placenta	Regulation of cell migration	1.30E-07	13
	Regulation of locomotion	5.18E-07	13
	Regulation of cell motion	5.47E-07	13
	Regulation of cell proliferation	1.15E-05	23
	Positive regulation of cell migration	3.08E-05	8
	Positive regulation of locomotion	5.75E-05	8
	Positive regulation of cell motion	5.75E-05	8
	Cell-substrate adhesion	5.75E-05	8
	Interphase of mitotic cell cycle	7.90E-05	8
	Interphase	9.47E-05	8
Esophagus	Oxidation reduction	9.42E-04	13
	Epidermis development	1.33E-03	7
	Ectoderm development	1.98E-03	7
	Cell projection assembly	2.17E-03	5

	Response to nutrient	2.29E-03	6
	Cell motion	3.97E-03	10
	Regulation of cell proliferation	5.29E-03	13
	Response to protein stimulus	5.40E-03	5
	Response to calcium ion	5.64E-03	4
	Cell junction organization	6.23E-03	4
B cells	Immune response	6.50E-06	16
	Positive regulation of immune system process	4.03E-04	8
	Regulation of T cell activation	5.34E-04	6
	Positive regulation of T cell activation	9.13E-04	5
	Lymphocyte activation	9.36E-04	7
	Regulation of lymphocyte activation	1.54E-03	6
	Positive regulation of lymphocyte activation	2.25E-03	5
	Leukocyte activation	2.53E-03	7
	Regulation of leukocyte activation	2.54E-03	6
	Regulation of T cell differentiation	3.03E-03	4
CD8+ T cells	Immune response	3.49E-02	8
	Chromatin modification	3.49E-02	5
	Hexose catabolic process	3.87E-02	3
	Monosaccharide catabolic process	4.07E-02	3
	Positive regulation of defense response	4.28E-02	3
	Alcohol catabolic process	5.16E-02	3
	Positive regulation of natural killer cell mediated cytotoxicity	6.98E-02	2
	Positive regulation of natural killer cell mediated immunity	6.98E-02	2
	Regulation of protein kinase activity	6.99E-02	5
	Response to drug	7.37E-02	4
Prostate	Muscle contraction	5.31E-04	6
	Muscle system process	8.11E-04	6
	Striated muscle contraction	1.05E-03	4
	Musculoskeletal movement	3.05E-03	3
	Multicellular organismal movement	3.05E-03	3
	Regulation of muscle contraction	3.80E-03	4
	Cytoskeleton organization	1.15E-02	7
	Muscle organ development	1.34E-02	5
	Segmentation	1.85E-02	3
	Actin filament-based process	2.08E-02	5
Colon	Carbohydrate biosynthetic process	5.36E-03	4
	Cellular carbohydrate biosynthetic process	2.20E-02	3
	Response to steroid hormone stimulus	2.58E-02	4
	Cellular response to nutrient	2.63E-02	2
	Response to nutrient levels	2.76E-02	4
	Response to glucocorticoid stimulus	2.77E-02	3

	Response to corticosteroid stimulus	3.24E-02	3
	Response to hormone stimulus	3.32E-02	5
	Response to drug	3.49E-02	4
	Response to extracellular stimulus	3.65E-02	4
Gut	Tissue remodeling	1.15E-03	4
	Cellular lipid catabolic process	2.76E-03	4
	Lipid catabolic process	3.76E-03	5
	Regulation of vasoconstriction	6.96E-03	3
	Angiotensin catabolic process in blood	7.38E-03	2
	Blood vessel morphogenesis	7.56E-03	5
	Protein digestion	1.10E-02	2
	Multicellular organismal macromolecule catabolic process	1.10E-02	2
	Multicellular organismal protein catabolic process	1.10E-02	2
	Blood vessel development	1.26E-02	5
CD4+ T cells	Translational elongation	7.81E-03	3
	Translation	9.02E-03	4
	Regulation of Ras protein signal transduction	3.12E-02	3
	Regulation of small GTPase mediated signal transduction	4.35E-02	3

Supplementary Table S2. Top 10 KEGG pathways significantly enriched with TSCTs for each of the 14 tissues. In this table, columns 1, 2, 3, 4 represent the tissue types, the most significantly enriched KEGG pathways, the enriched p-values, and the numbers of TSCTs involved in the KEGG pathways, respectively. All the KEGG pathways were listed if the number of enriched KEGG pathways under 10, and no KEGG pathway was enriched with TSCTs of CD4+ T cells.

Tissue type	Biological process	P-value	Count
Testis	Basal transcription factors	2.31E-03	7
	O-Mannosyl glycan biosynthesis	4.63E-03	3
	Ubiquitin mediated proteolysis	8.16E-03	13
	Proteasome	1.03E-02	7
	Cell cycle	1.08E-02	12
	Glycerophospholipid metabolism	1.79E-02	8
	mTOR signaling pathway	5.50E-02	6
	Oocyte meiosis	7.16E-02	9
	RNA degradation	7.57E-02	6
Brain	Axon guidance	1.97E-06	17
	Gap junction	3.00E-06	14
	Pathogenic Escherichia coli infection	8.19E-06	11
	Oocyte meiosis	2.14E-03	11
	Endocytosis	4.69E-03	14
	Long-term depression	5.43E-03	8
	Neurotrophin signaling pathway	1.50E-02	10
	Epithelial cell signaling in Helicobacter pylori infection	1.88E-02	7
	MAPK signaling pathway	1.90E-02	16
	Alzheimer's disease	6.82E-02	10
Monocytes	Lysosome	4.26E-06	15
	Chemokine signaling pathway	1.70E-05	18
	Toll-like receptor signaling pathway	1.14E-04	12
	Leukocyte transendothelial migration	1.73E-03	11
	Pathogenic Escherichia coli infection	5.14E-03	7
	Fc gamma R-mediated phagocytosis	1.75E-02	8
	Cytosolic DNA-sensing pathway	1.90E-02	6
	NOD-like receptor signaling pathway	3.02E-02	6
	Pentose phosphate pathway	3.24E-02	4
	Hematopoietic cell lineage	3.42E-02	7
Ovary	DNA replication	2.75E-04	6
	Cell cycle	9.71E-04	9
	Basal transcription factors	2.46E-03	5

	Progesterone-mediated oocyte maturation	2.78E-03	7
	Ubiquitin mediated proteolysis	7.12E-03	8
	Base excision repair	1.95E-02	4
	Nucleotide excision repair	3.54E-02	4
	Mismatch repair	5.45E-02	3
	Pyrimidine metabolism	7.12E-02	5
Heart	Dilated cardiomyopathy	1.33E-07	11
	Hypertrophic cardiomyopathy (HCM)	7.53E-07	10
	Cardiac muscle contraction	4.23E-05	8
	Parkinson's disease	9.31E-04	8
	Huntington's disease	1.54E-03	9
	Arrhythmogenic right ventricular cardiomyopathy (ARVC)	2.34E-03	6
	Alzheimer's disease	3.74E-03	8
	Oxidative phosphorylation	5.12E-03	7
	Glycolysis / Gluconeogenesis	3.76E-02	4
	Fructose and mannose metabolism	6.58E-02	3
Liver	Steroid biosynthesis	5.25E-09	8
	PPAR signaling pathway	1.74E-05	9
	Valine, leucine and isoleucine degradation	8.28E-05	7
	Terpenoid backbone biosynthesis	9.03E-05	5
	Porphyrin and chlorophyll metabolism	2.01E-04	6
	Biosynthesis of unsaturated fatty acids	4.42E-04	5
	Fatty acid metabolism	5.08E-04	6
	Glycine, serine and threonine metabolism	1.69E-03	5
	Butanoate metabolism	2.40E-03	5
	Complement and coagulation cascades	5.92E-03	6
Placenta	ECM-receptor interaction	7.78E-09	12
	Focal adhesion	2.23E-07	15
	Small cell lung cancer	1.02E-04	8
	Pathways in cancer	2.78E-04	14
	p53 signaling pathway	1.19E-02	5
	Prostate cancer	2.91E-02	5
	Complement and coagulation cascades	6.19E-02	4
	Renal cell carcinoma	6.41E-02	4
	Cell cycle	8.17E-02	5
	Sphingolipid metabolism	9.28E-02	3
Esophagus	Glycerolipid metabolism	5.84E-03	4
	ECM-receptor interaction	3.14E-02	4
	Metabolism of xenobiotics by cytochrome P450	8.73E-02	3
B cells	Intestinal immune network for IgA production	2.54E-04	5
	Fc gamma R-mediated phagocytosis	3.21E-04	6
	B cell receptor signaling pathway	1.29E-03	5
	Phosphatidylinositol signaling system	1.18E-02	4

	Systemic lupus erythematosus	2.56E-02	4
	Graft-versus-host disease	2.61E-02	3
	Type I diabetes mellitus	2.99E-02	3
	Leukocyte transendothelial migration	4.01E-02	4
	Autoimmune thyroid disease	4.27E-02	3
	Neurotrophin signaling pathway	4.54E-02	4
CD8+ T cells	Ribosome	2.24E-03	5
	Glycolysis / Gluconeogenesis	5.72E-02	3
Prostate	Hypertrophic cardiomyopathy (HCM)	5.57E-02	3
	Dilated cardiomyopathy	6.41E-02	3
	Vascular smooth muscle contraction	9.01E-02	3
Colon	Amino sugar and nucleotide sugar metabolism	2.24E-02	3
Gut	Renin-angiotensin system	6.02E-05	4
	Arginine and proline metabolism	2.54E-02	3

Supplementary Table S3. Sample information.

No.	Tissue or Cell Type	Donor	Total RNA (ug)	Conc. (ng/ul)	Vol. (ul)	28S/18S	RIN
1	Colon	A	5	598	8.3	1.6	6.8
2	Esophagus	A	5	879	5.6	1.1	6.8
3	Prostate	A	5	368	13.5	0.9	8.6
4	Testes	A	5	2220	2.25	1.4	6.2
5	B cells	B	2	49	40.8	2.3	10
6	Monocytes	B	5	654	7.6	1.4	9.1
7	CD4+ T cells (memory)	B	5	538	9.3	2.5	7.4
8	CD8+ T cells	B	5	281	17.8	2.5	9.8
9	Fetal Brain	E	5	326	15.3	1.8	9.3
10	Fetal Gut	E	2	29	68..9	2.4	8.6
11	Fetal Heart	E	2	44	45.4	1.4	9.4
12	Fetal Liver	E	5	183	10.9	1.2	8.6
13	Fetal Ovary	E	5	223	22.4	2.1	9.8
14	Placenta	E	5	129	38.8	1.2	6.8
